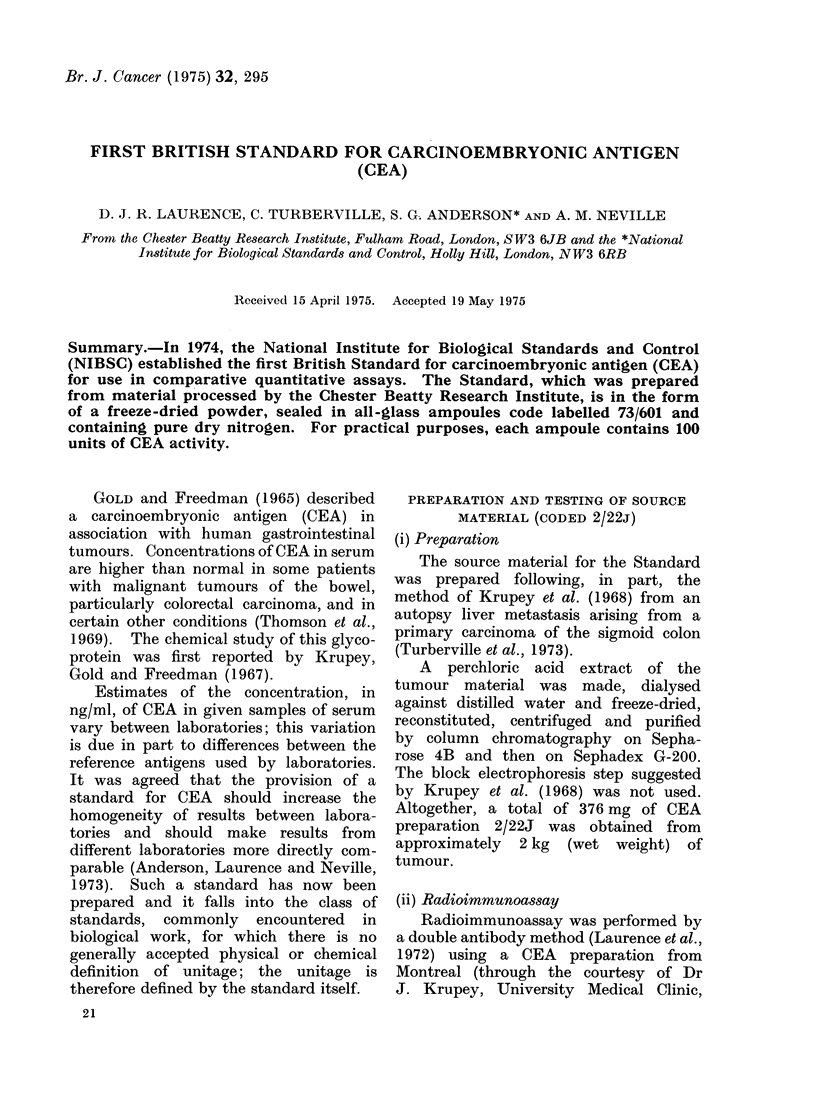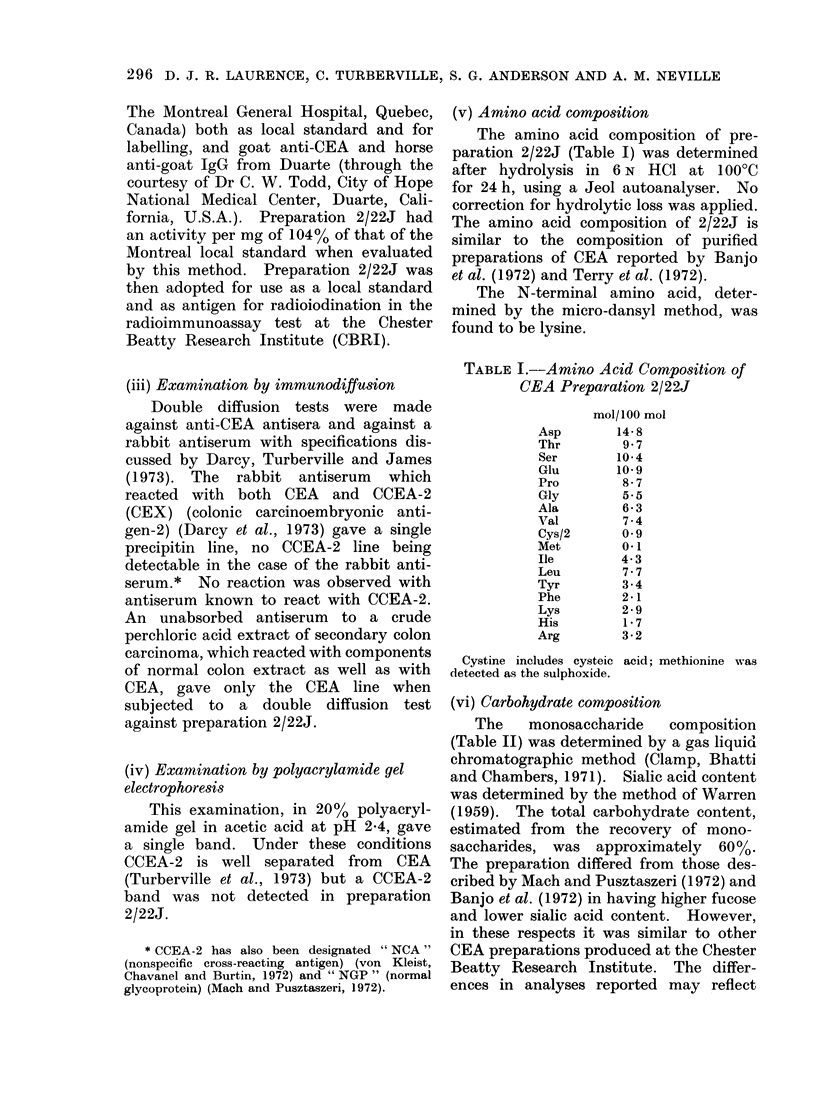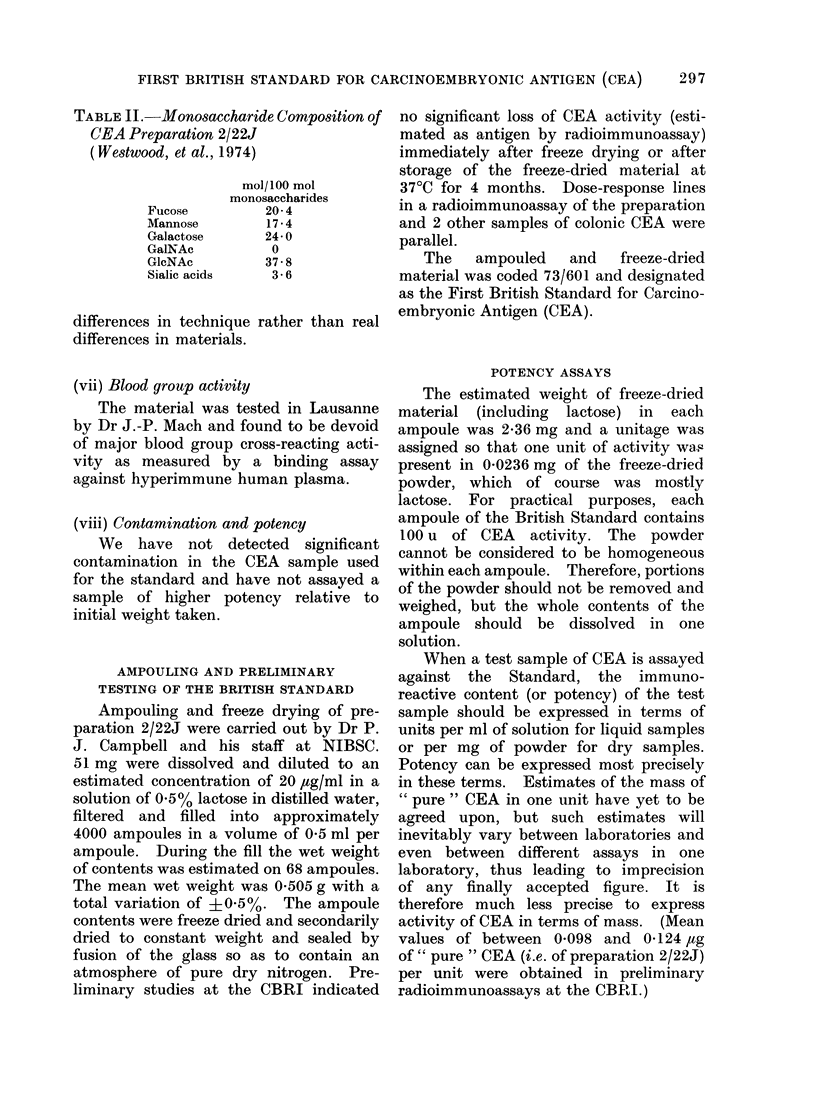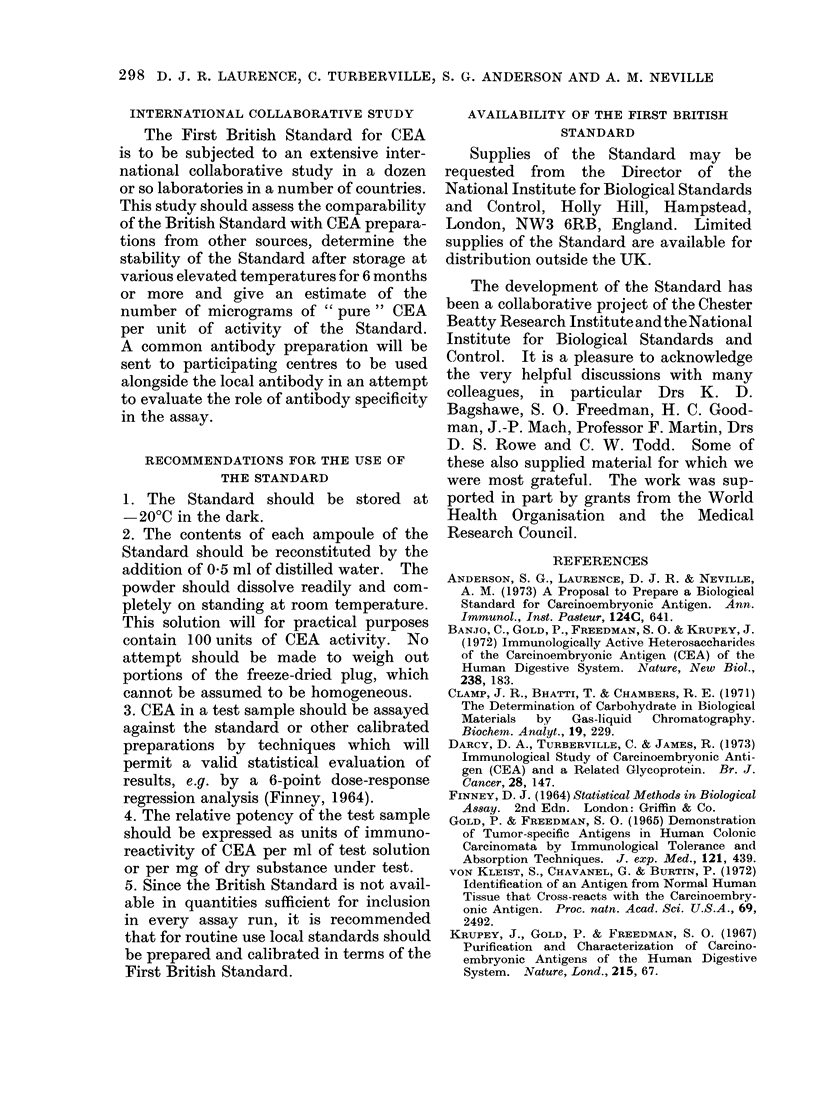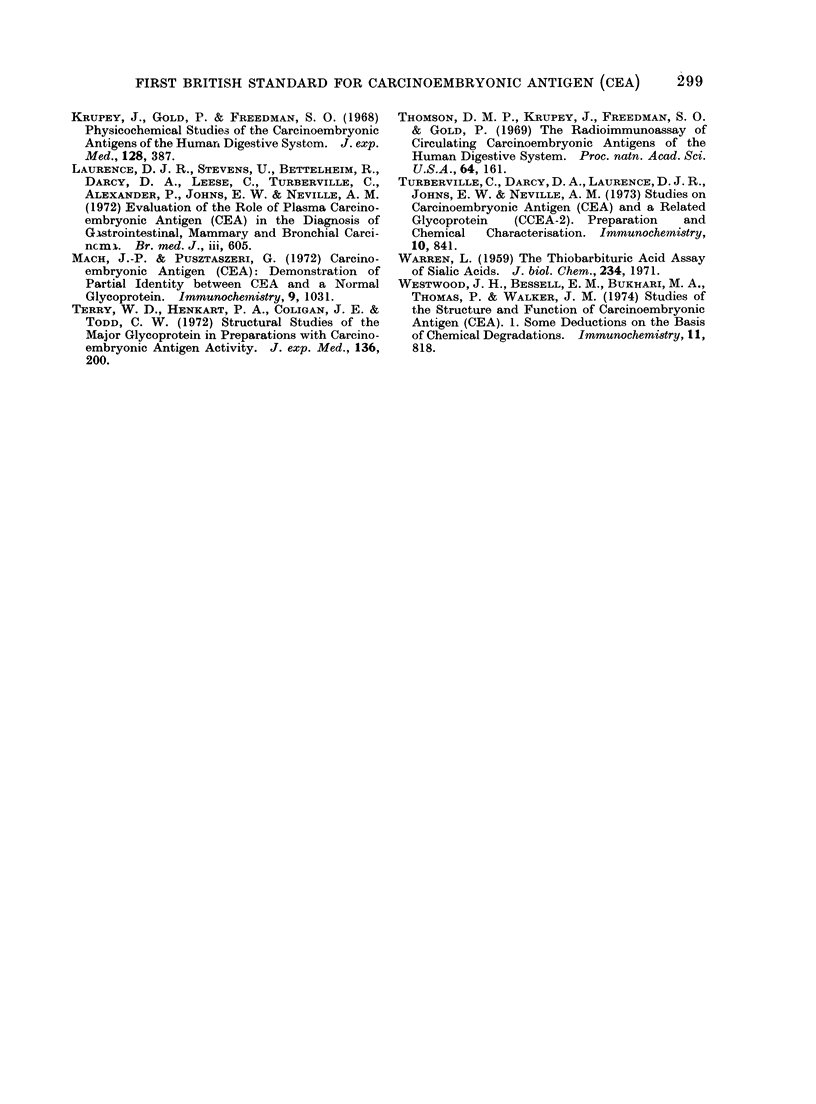# First British standard for carcinoembryonic antigen (CEA).

**DOI:** 10.1038/bjc.1975.227

**Published:** 1975-09

**Authors:** D. J. Laurence, C. Turberville, S. G. Anderson, A. M. Neville

## Abstract

In 1974, the National Institute for Biological Standards and Control (NIBSC) established the first British Standard for carcinoembryonic antigen (CEA) for use in comparative quantitative assays. The Standard, which was prepared for material processed by the Chester Beatty Research Institute, is in the form of a freeze-dried powder, sealed in all glass ampoules code labelled 73/601 and containing pure dry nitrogen. For practical purposes, each ampoule contains 100 units of CEA activity.


*Br. J. Cancer* (1975) **32**, 295

### FIRST BRITISH STANDARD FOR CARCINOEMBRYONIC ANTIGEN (CEA)

D. J. R. LAURENCE, C. TURBERVILLE, S. G. ANDERSON* AND A. M. NEVILLE

*From the Chester Beatty Research Institute, Fulham Road, London, SW3 6JB and the *National Institute for Biological Standards and Control, Holly Hill, London, NW3 6RB*

Received 15 April 1975. Accepted 19 May 1975

**Summary.—In 1974, the National Institute for Biological Standards and Control (NIBSC) established the first British Standard for carcinoembryonic antigen (CEA) for use in comparative quantitative assays. The Standard, which was prepared from material processed by the Chester Beatty Research Institute, is in the form of a freeze-dried powder, sealed in all-glass ampoules code labelled 73/601 and containing pure dry nitrogen. For practical purposes, each ampoule contains 100 units of CEA activity.**

GOLD and Freedman (1965) described a carcinoembryonic antigen (CEA) in association with human gastrointestinal tumours. Concentrations of CEA in serum are higher than normal in some patients with malignant tumours of the bowel, particularly colorectal carcinoma, and in certain other conditions (Thomson *et al.*, 1969). The chemical study of this glycoprotein was first reported by Krupey, Gold and Freedman (1967).

Estimates of the concentration, in ng/ml, of CEA in given samples of serum vary between laboratories; this variation is due in part to differences between the reference antigens used by laboratories. It was agreed that the provision of a standard for CEA should increase the homogeneity of results between laboratories and should make results from different laboratories more directly comparable (Anderson, Laurence and Neville, 1973). Such a standard has now been prepared and it falls into the class of standards, commonly encountered in biological work, for which there is no generally accepted physical or chemical definition of unitage; the unitage is therefore defined by the standard itself.

### PREPARATION AND TESTING OF SOURCE MATERIAL (CODED 2/22J)

### (i) *Preparation*

The source material for the Standard was prepared following, in part, the method of Krupey *et al.* (1968) from an autopsy liver metastasis arising from a primary carcinoma of the sigmoid colon (Turberville *et al.*, 1973).

A perchloric acid extract of the tumour material was made, dialysed against distilled water and freeze-dried, reconstituted, centrifuged and purified by column chromatography on Sepharose 4B and then on Sephadex G-200. The block electrophoresis step suggested by Krupey *et al.* (1968) was not used. Altogether, a total of 376 mg of CEA preparation 2/22J was obtained from approximately 2 kg (wet weight) of tumour.

### (ii) *Radioimmunoassay*

Radioimmunoassay was performed by a double antibody method (Laurence *et al.*, 1972) using a CEA preparation from Montreal (through the courtesy of Dr J. Krupey, University Medical Clinic,

The Montreal General Hospital, Quebec, Canada) both as local standard and for labelling, and goat anti-CEA and horse anti-goat IgG from Duarte (through the courtesy of Dr C. W. Todd, City of Hope National Medical Center, Duarte, California, U.S.A.). Preparation 2/22J had an activity per mg of 104% of that of the Montreal local standard when evaluated by this method. Preparation 2/22J was then adopted for use as a local standard and as antigen for radioiodination in the radioimmunoassay test at the Chester Beatty Research Institute (CBRI).

(iii) *Examination by immunodiffusion*

Double diffusion tests were made against anti-CEA antisera and against a rabbit antiserum with specifications discussed by Darcy, Turberville and James (1973). The rabbit antiserum which reacted with both CEA and CCEA-2 (CEX) (colonic carcinoembryonic antigen-2) (Darcy *et al.*, 1973) gave a single precipitin line, no CCEA-2 line being detectable in the case of the rabbit antiserum.* No reaction was observed with antiserum known to react with CCEA-2. An unabsorbed antiserum to a crude perchloric acid extract of secondary colon carcinoma, which reacted with components of normal colon extract as well as with CEA, gave only the CEA line when subjected to a double diffusion test against preparation 2/22J.

(iv) *Examination by polyacrylamide gel electrophoresis*

This examination, in 20% polyacrylamide gel in acetic acid at pH 2·4, gave a single band. Under these conditions CCEA-2 is well separated from CEA (Turberville *et al.*, 1973) but a CCEA-2 band was not detected in preparation 2/22J.

* CCEA-2 has also been designated " NCA " (nonspecific cross-reacting antigen) (von Kleist, Chavanel and Burtin, 1972) and " NGP " (normal glycoprotein) (Mach and Pusztaszeri, 1972).

(v) *Amino acid composition*

The amino acid composition of preparation 2/22J (Table I) was determined after hydrolysis in 6 N HCl at 100°C for 24 h, using a Jeol autoanalyser. No correction for hydrolytic loss was applied. The amino acid composition of 2/22J is similar to the composition of purified preparations of CEA reported by Banjo *et al.* (1972) and Terry *et al.* (1972).

The N-terminal amino acid, determined by the micro-dansyl method, was found to be lysine.

TABLE I.—*Amino Acid Composition of CEA Preparation 2/22J*

| | mol/100 mol |
|---|---|
| Asp | 14·8 |
| Thr | 9·7 |
| Ser | 10·4 |
| Glu | 10·9 |
| Pro | 8·7 |
| Gly | 5·5 |
| Ala | 6·3 |
| Val | 7·4 |
| Cys/2 | 0·9 |
| Met | 0·1 |
| Ile | 4·3 |
| Leu | 7·7 |
| Tyr | 3·4 |
| Phe | 2·1 |
| Lys | 2·9 |
| His | 1·7 |
| Arg | 3·2 |

Cystine includes cysteic acid; methionine was detected as the sulphoxide.

(vi) *Carbohydrate composition*

The monosaccharide composition (Table II) was determined by a gas liquid chromatographic method (Clamp, Bhatti and Chambers, 1971). Sialic acid content was determined by the method of Warren (1959). The total carbohydrate content, estimated from the recovery of monosaccharides, was approximately 60%. The preparation differed from those described by Mach and Pusztaszeri (1972) and Banjo *et al.* (1972) in having higher fucose and lower sialic acid content. However, in these respects it was similar to other CEA preparations produced at the Chester Beatty Research Institute. The differences in analyses reported may reflect

TABLE II.—*Monosaccharide Composition of CEA Preparation* 2/22J (*Westwood, et al.*, 1974)

| | mol/100 mol monosaccharides |
|---|---|
| Fucose | 20·4 |
| Mannose | 17·4 |
| Galactose | 24·0 |
| GalNAc | 0 |
| GlcNAc | 37·8 |
| Sialic acids | 3·6 |

differences in technique rather than real differences in materials.

(vii) *Blood group activity*

The material was tested in Lausanne by Dr J.-P. Mach and found to be devoid of major blood group cross-reacting activity as measured by a binding assay against hyperimmune human plasma.

(viii) *Contamination and potency*

We have not detected significant contamination in the CEA sample used for the standard and have not assayed a sample of higher potency relative to initial weight taken.

### AMPOULING AND PRELIMINARY TESTING OF THE BRITISH STANDARD

Ampouling and freeze drying of preparation 2/22J were carried out by Dr P. J. Campbell and his staff at NIBSC. 51 mg were dissolved and diluted to an estimated concentration of 20 $\mu$g/ml in a solution of 0·5% lactose in distilled water, filtered and filled into approximately 4000 ampoules in a volume of 0·5 ml per ampoule. During the fill the wet weight of contents was estimated on 68 ampoules. The mean wet weight was 0·505 g with a total variation of $\pm$0·5%. The ampoule contents were freeze dried and secondarily dried to constant weight and sealed by fusion of the glass so as to contain an atmosphere of pure dry nitrogen. Preliminary studies at the CBRI indicated no significant loss of CEA activity (estimated as antigen by radioimmunoassay) immediately after freeze drying or after storage of the freeze-dried material at 37°C for 4 months. Dose-response lines in a radioimmunoassay of the preparation and 2 other samples of colonic CEA were parallel.

The ampouled and freeze-dried material was coded 73/601 and designated as the First British Standard for Carcinoembryonic Antigen (CEA).

### POTENCY ASSAYS

The estimated weight of freeze-dried material (including lactose) in each ampoule was 2·36 mg and a unitage was assigned so that one unit of activity was present in 0·0236 mg of the freeze-dried powder, which of course was mostly lactose. For practical purposes, each ampoule of the British Standard contains 100 u of CEA activity. The powder cannot be considered to be homogeneous within each ampoule. Therefore, portions of the powder should not be removed and weighed, but the whole contents of the ampoule should be dissolved in one solution.

When a test sample of CEA is assayed against the Standard, the immunoreactive content (or potency) of the test sample should be expressed in terms of units per ml of solution for liquid samples or per mg of powder for dry samples. Potency can be expressed most precisely in these terms. Estimates of the mass of " pure " CEA in one unit have yet to be agreed upon, but such estimates will inevitably vary between laboratories and even between different assays in one laboratory, thus leading to imprecision of any finally accepted figure. It is therefore much less precise to express activity of CEA in terms of mass. (Mean values of between 0·098 and 0·124 $\mu$g of " pure " CEA (*i.e.* of preparation 2/22J) per unit were obtained in preliminary radioimmunoassays at the CBRI.)

### INTERNATIONAL COLLABORATIVE STUDY

The First British Standard for CEA is to be subjected to an extensive international collaborative study in a dozen or so laboratories in a number of countries. This study should assess the comparability of the British Standard with CEA preparations from other sources, determine the stability of the Standard after storage at various elevated temperatures for 6 months or more and give an estimate of the number of micrograms of "pure" CEA per unit of activity of the Standard. A common antibody preparation will be sent to participating centres to be used alongside the local antibody in an attempt to evaluate the role of antibody specificity in the assay.

### RECOMMENDATIONS FOR THE USE OF THE STANDARD

1. The Standard should be stored at −20°C in the dark.
2. The contents of each ampoule of the Standard should be reconstituted by the addition of 0·5 ml of distilled water. The powder should dissolve readily and completely on standing at room temperature. This solution will for practical purposes contain 100 units of CEA activity. No attempt should be made to weigh out portions of the freeze-dried plug, which cannot be assumed to be homogeneous.
3. CEA in a test sample should be assayed against the standard or other calibrated preparations by techniques which will permit a valid statistical evaluation of results, *e.g.* by a 6-point dose-response regression analysis (Finney, 1964).
4. The relative potency of the test sample should be expressed as units of immunoreactivity of CEA per ml of test solution or per mg of dry substance under test.
5. Since the British Standard is not available in quantities sufficient for inclusion in every assay run, it is recommended that for routine use local standards should be prepared and calibrated in terms of the First British Standard.

### AVAILABILITY OF THE FIRST BRITISH STANDARD

Supplies of the Standard may be requested from the Director of the National Institute for Biological Standards and Control, Holly Hill, Hampstead, London, NW3 6RB, England. Limited supplies of the Standard are available for distribution outside the UK.

The development of the Standard has been a collaborative project of the Chester Beatty Research Institute and the National Institute for Biological Standards and Control. It is a pleasure to acknowledge the very helpful discussions with many colleagues, in particular Drs K. D. Bagshawe, S. O. Freedman, H. C. Goodman, J.-P. Mach, Professor F. Martin, Drs D. S. Rowe and C. W. Todd. Some of these also supplied material for which we were most grateful. The work was supported in part by grants from the World Health Organisation and the Medical Research Council.

### REFERENCES

ANDERSON, S. G., LAURENCE, D. J. R. & NEVILLE, A. M. (1973) A Proposal to Prepare a Biological Standard for Carcinoembryonic Antigen. *Ann. Immunol., Inst. Pasteur*, **124C**, 641.

BANJO, C., GOLD, P., FREEDMAN, S. O. & KRUPEY, J. (1972) Immunologically Active Heterosaccharides of the Carcinoembryonic Antigen (CEA) of the Human Digestive System. *Nature, New Biol.*, **238**, 183.

CLAMP, J. R., BHATTI, T. & CHAMBERS, R. E. (1971) The Determination of Carbohydrate in Biological Materials by Gas-liquid Chromatography. *Biochem. Analyt.*, **19**, 229.

DARCY, D. A., TURBERVILLE, C. & JAMES, R. (1973) Immunological Study of Carcinoembryonic Antigen (CEA) and a Related Glycoprotein. *Br. J. Cancer*, **28**, 147.

FINNEY, D. J. (1964) *Statistical Methods in Biological Assay.* 2nd Edn. London: Griffin & Co.

GOLD, P. & FREEDMAN, S. O. (1965) Demonstration of Tumor-specific Antigens in Human Colonic Carcinomata by Immunological Tolerance and Absorption Techniques. *J. exp. Med.*, **121**, 439.

VON KLEIST, S., CHAVANEL, G. & BURTIN, P. (1972) Identification of an Antigen from Normal Human Tissue that Cross-reacts with the Carcinoembryonic Antigen. *Proc. natn. Acad. Sci. U.S.A.*, **69**, 2492.

KRUPEY, J., GOLD, P. & FREEDMAN, S. O. (1967) Purification and Characterization of Carcinoembryonic Antigens of the Human Digestive System. *Nature, Lond.*, **215**, 67.

Krupey, J., Gold, P. & Freedman, S. O. (1968) Physicochemical Studies of the Carcinoembryonic Antigens of the Human Digestive System. *J. exp. Med.*, **128**, 387.

Laurence, D. J. R., Stevens, U., Bettelheim, R., Darcy, D. A., Leese, C., Turberville, C., Alexander, P., Johns, E. W. & Neville, A. M. (1972) Evaluation of the Role of Plasma Carcinoembryonic Antigen (CEA) in the Diagnosis of Gastrointestinal, Mammary and Bronchial Carcinoma. *Br. med. J.*, iii, 605.

Mach, J.-P. & Pusztaszeri, G. (1972) Carcinoembryonic Antigen (CEA): Demonstration of Partial Identity between CEA and a Normal Glycoprotein. *Immunochemistry*, **9**, 1031.

Terry, W. D., Henkart, P. A., Coligan, J. E. & Todd, C. W. (1972) Structural Studies of the Major Glycoprotein in Preparations with Carcinoembryonic Antigen Activity. *J. exp. Med.*, **136**, 200.

Thomson, D. M. P., Krupey, J., Freedman, S. O. & Gold, P. (1969) The Radioimmunoassay of Circulating Carcinoembryonic Antigens of the Human Digestive System. *Proc. natn. Acad. Sci. U.S.A.*, **64**, 161.

Turberville, C., Darcy, D. A., Laurence, D. J. R., Johns, E. W. & Neville, A. M. (1973) Studies on Carcinoembryonic Antigen (CEA) and a Related Glycoprotein (CCEA-2). Preparation and Chemical Characterisation. *Immunochemistry*, **10**, 841.

Warren, L. (1959) The Thiobarbituric Acid Assay of Sialic Acids. *J. biol. Chem.*, **234**, 1971.

Westwood, J. H., Bessell, E. M., Bukhari, M. A., Thomas, P. & Walker, J. M. (1974) Studies of the Structure and Function of Carcinoembryonic Antigen (CEA). 1. Some Deductions on the Basis of Chemical Degradations. *Immunochemistry*, **11**, 818.